# Polyols and UV‐sunscreens in the *Prasiola*‐clade (Trebouxiophyceae, Chlorophyta) as metabolites for stress response and chemotaxonomy

**DOI:** 10.1111/jpy.12619

**Published:** 2018-02-21

**Authors:** Vivien Hotter, Karin Glaser, Anja Hartmann, Markus Ganzera, Ulf Karsten

**Affiliations:** ^1^ Institute of Biological Sciences, Applied Ecology and Phycology University of Rostock Albert‐Einstein‐Straße 3 D‐18059 Rostock Germany; ^2^ Institute of Pharmacy, Pharmacognosy University of Innsbruck Innrain 80‐82/IV A‐6020 Innsbruck Austria

**Keywords:** chemotaxonomy, MAAs, polyols, prasiolin, sunscreen, terrestrial algae, UV radiation

## Abstract

In many regions of the world, aeroterrestrial green algae of the Trebouxiophyceae (Chlorophyta) represent very abundant soil microorganisms, and hence their taxonomy is crucial to investigate their physiological performance and ecological importance. Due to a lack in morphological features, taxonomic and phylogenetic studies of Trebouxiophycean algae can be a challenging task. Since chemotaxonomic markers could be a great assistance in this regard, 22 strains of aeroterrestrial Trebouxiophyceae were chemically screened for their polyol‐patterns as well as for mycosporine‐like amino acids (MAAs) in their aqueous extracts using RP‐HPLC and LC‐MS. d‐sorbitol was exclusively detected in members of the Prasiolaceae family. The novel MAA prasiolin and a related compound (“prasiolin‐like”) were present in all investigated members of the *Prasiola*‐clade, but missing in all other tested Trebouxiophyceae. While prasiolin could only be detected in field material directly after extraction, the “prasiolin‐like” compound present in the other algae was fully converted into prasiolin after 24 h. These findings suggest d‐sorbitol and prasiolin‐like compounds are suitable chemotaxonomic markers for the Prasiolaceae and *Prasiola*‐clade, respectively. Additional UV‐exposure experiments with selected strains show that MAA formation and accumulation can be induced, supporting their role as UV‐sunscreen.

**Abbreviations**
MAAs: mycosporine‐like amino acids
RP‐HPLC: reversed phase high performance liquid chromatography
UVR: ultraviolet radiation

In contrast to their aquatic relatives, aeroterrestrial algae are directly exposed to the atmosphere and thereby subject to harsh environmental conditions, such as strong diurnal and seasonal changes of ultraviolet radiation (UVR; Hartmann et al. 2016) and strong differences between cellular and atmospheric water potential (Holzinger and Karsten 2013). Differences in water potential drive water movement across membranes, between different cellular compartments, and between organisms and their environment (e.g., Larcher 2003), and thus determine, for example, water availability for poikilohydric organisms, such as green algae (Kranner et al. 2008). Due to the incapability of green algae to actively regulate the water budget, their cells desiccate if the extracellular water potential is lower than the intracellular one. Since life without water is impossible, uncontrolled dehydration leads to increasing mortality unless an organism is desiccation tolerant. In arid regions water is scarce and its availability unpredictable. Nevertheless, many members of the Chlorophyta and Streptophyta such as *Interfilum*,* Klebsormidium*,* Coccomyxa*,* Rosenvingiella*, or *Prasiola* are found in various terrestrial habitats all over the world, from deserts and alpine regions to urban areas, where they inhabit both natural and artificial surfaces such as soil, tree bark, roof tiles, etc. (e.g., Karsten et al. 2007a,b,c, Rindi 2007, Moniz et al. 2012, Darienko et al. 2015, Ryšánek et al. 2015, and references therein).

Aeroterrestrial green algae developed numerous mechanisms to survive desiccation (for review see Holzinger and Karsten 2013). Green algal members of the class Trebouxiophyceae are capable of synthesizing and accumulating polyols. These low molecular weight carbohydrates exhibit multiple functions. They act as antioxidants, stabilize proteins under heat stress conditions and are rapidly available respiratory substrates in case of energy deficiency (Yancey 2005, Karsten et al. 2007c). Polyols are also osmotically active, that is, they decrease the intracellular water potential when accumulated (Holzinger and Karsten 2013). Thereby, desiccation is reduced or even prevented without negatively affecting metabolic integrity. Hence, polyols are also called compatible solutes (Yancey 2005). Aeroterrestrial members of the Trebouxiophyceae, such as *Apatococcus*,* Chloroidium*,* Coccomyxa*,* Prasiola*,* Rosenvingiella*,* Stichococcus* and *Trentepohlia*, synthesize a variety of polyols, such as arabitol, erythritol, glycerol, ribitol, D‐sorbitol and volemitol (Feige and Kremer 1980, Gustavs et al. 2010, 2011). While in some clades like *Apatococcus* spp. a combination of these substances can be detected, others like *Prasiola* spp. contain only one compound (Gustavs et al. 2011). Therefore, polyol and other low molecular weight carbohydrate patterns can be used for chemotaxonomy (Karsten et al. 1999).

Throughout the day or season, exposure to UVR can change rapidly. While UV‐C (200–280 nm) is biologically irrelevant as this wavelength is absorbed by the ozone layer of the stratosphere, both UV‐A (315–400 nm) and UV‐B (280–315 nm) reach earth's surface. UV‐B radiation is especially harmful to many biological processes (McKenzie et al. 2007, and references therein), such as photosynthesis or enzyme activity (Holzinger and Lütz 2006, Sharma et al. 2017, and references therein). Since photosynthesis is a physiological key process for algae, its function is vital.

To oppose UVR damage, aeroterrestrial Trebouxiophyceae belonging to the *Lobosphaera*‐, *Watanabea*‐ and *Prasiola*‐clade biosynthesize and accumulate mycosporine‐like amino acids (MAAs; Karsten et al. 2005, 2007b). These sunscreen compounds absorb UVR and re‐emit it as harmless heat, thereby shielding intracellular structures and biomolecules (Bandaranayake 1998). MAAs are the most common photoprotective compounds in aquatic organisms, from cyanobacteria and algae to invertebrates and fish (Dunlap and Shick 1998, Sommaruga and Garcia‐Pichel 1999). While MAAs have been investigated extensively in red algae (Karsten et al. 1998, Franklin et al. 1999, Karsten and Wiencke 1999, Karsten 2000, Kräbs et al. 2002, Boedeker and Karsten 2005, Pandey et al. 2017) as well as in cyanobacteria and lichens (Garcia‐Pichel et al. 1993, Budel et al. 1997, Pattanaik et al. 2008, Hartmann et al. 2015), only little is known about their presence in aeroterrestrial Trebouxiophyceae and even less in the *Prasiola*‐clade (Hartmann et al. 2015). A putative MAA within the Trebouxiophyceae was first found in *Prasiola crispa* ssp. *antarctica* by Hoyer et al. (2001). Using High Performance Liquid Chromatography (HPLC), a unique UV‐absorbing compound with an absorption maximum at 324 nm was described. Groeniger and Haeder (2002) not only confirmed this putative 324 nm‐MAA in the closely related *Prasiola stipitata*, but also proved its inducibility by UV exposure. A chemical screening of various members of the Trebouxiophyceae confirmed the occurrence of this 324 nm‐MAA in *Watanabea* spp. and *Prasiola* spp. (Karsten et al. 2005, 2007b). Recently, Hartmann et al. (2016) elucidated the chemical structure of this putative 324 nm‐MAA in the closely related *Prasiola calophylla* as *N*‐[5,6 hydroxy‐5(hydroxymethyl)‐2‐methoxy‐3‐oxo‐1‐cycohexen‐1‐yl] glutamic acid, which indeed represents a novel MAA. It was named prasiolin. However, so far only a few members of the Trebouxiophyceae have been studied for the presence of this and other MAAs and until now the occurrence of prasiolin is experimentally proven only in *P. calophylla*.

Stress metabolites such as polyols and MAAs are not only essential for the long‐term survival of green algae under atmospheric conditions, but they can also be useful in chemotaxonomy. Many aeroterrestrial green algae resemble each other morphologically (Rindi 2007) and some species even display high phenotypic plasticity (Rindi and Guiry 2002, Darienko et al. 2015, 2016). This makes green algal identification down to the species level based on morphological traits often complicated and sometimes even unreliable (John and Maggs 1997, Rindi 2007). Polyphasic approaches combining morphological, molecular and/or physiological/biochemical data sets are a promising solution to overcome these problems in species identification (Proeschold and Leliaert 2007, Coesel and Krienitz 2008, Darienko et al. 2010).

In chemotaxonomy, chemical traits are used to assign organisms to taxa with equal compounds. Any chemical compound is suitable as a chemotaxonomic marker if it is taxon specific, consistent within a lineage and present in detectable amounts (Karsten et al. 2007a). Prominent examples are photosynthetic pigments for algae subdivision (Roy et al. 2011) or low‐molecular weight carbohydrate patterns to distinguish lineages within the Bangiophyceae (Karsten et al. 1999). Stress metabolites can also be suitable chemotaxonomic markers (Darienko et al. 2010). Gustavs et al. (2011) screened a wide range of Trebouxiophyceae for their polyols and detected several clade‐specific patterns. For instance, d‐sorbitol was proposed as a marker for the *Prasiola*‐, d‐ribitol for the *Watanabea*‐ and a combination of d‐ribitol and erythritol for the *Apatococcus*‐clade (Gustavs et al. 2011).

In this study, 22 strains of aeroterrestrial Trebouxiophyceae were examined for their polyol patterns and the presence of MAAs using HPLC. All strains were chosen due to their abundance in terrestrial habitats, such as biofilms or soil (Jacob et al. 1991, Rindi and Guiry 2004) with a focus on species related to *P. calophylla* (Hartmann et al. 2016). Latest green algal phylogenies were used as a reference (Hallmann et al. 2016, Hodač et al. 2016, Garrido‐Benavent et al. 2017, Richter et al. 2017). Based on the findings of Gustavs et al. (2011), we hypothesized d‐sorbitol to be a suitable chemotaxonomical marker for the *Prasiola*‐clade. Since UVR is a regular stressor for aeroterrestrial green algae and because prasiolin was recently identified in *P. calophylla* (Hartmann et al. 2016), this and chemically similar MAAs were expected in all members of the *Prasiola*‐clade. Additionally, UVR exposure experiments were conducted for some selected Trebouxiophyceae strains to test the induction of MAAs as a UV protective mechanism.

## Materials and Methods

### Algal material and culture conditions

A total of 22 aeroterrestrial Trebouxiophyceae strains were chemically screened for the presence of both polyols and MAAs: 16 unialgal cultures from the Sammlung von Algenkulturen at the University of Göttingen, Germany (SAG), two unialgal cultures from the Station Biologique de Roscoff, France, and four field samples (Table S1 in the Supporting Information). Strains from Roscoff were grown in Provasoli‐enriched full‐strength seawater (Provasoli 1968) at 13°C for 10 weeks, irradiated with 40 μmol photons · m^−2^ · s^−1^ (Lumilux Cool Daylight L18W/865; OSRAM, Munich, Germany) under a 12:12 h light:dark cycle. Algal biomass was dried in silica gel prior to polyol and MAA extraction. All SAG strains were grown in 50 mL Erlenmeyer flasks filled with modified Bold's basal medium (Starr and Zeikus 1993) for 16 d at a temperature of 20°C. Daylight lamps (Lumilux Deluxe Daylight L15W/950; OSRAM) emitted PAR with a photon flux density of 25–30 μmol photons · m^−2^ · s^−1^ under a 16:8 h light:dark cycle. Afterwards, algal biomass was harvested by filtration (GF 6 filters; Carl Roth, Karlsruhe, Germany) and dried at 30°C overnight. *Prasiola calophylla* field material was collected by Dr. Andreas Holzinger at the Botanical Garden, University of Innsbruck, Austria, in January 2017 and lyophilized. *Rosenvingiella radicans* (SRN 75) was collected by Dr. Svenja Heesch in Bodø, Norway, in October 2016 and dried in silica gel. *Prasiola stipitata* was collected by Dr. S. Heesch in Roscoff, France, in February 2017 and dried in silica gel, too (Table 1). Dry weight (DW) was always determined for all algae samples prior polyol and MAA extraction.

**Table 1:** Prasiolin/“prasiolin‐like,” d‐sorbitol and d‐ribitol content detected in the investigated Trebouxiophyceae in mg · g^−1^ dry weight (DW). All investigated members of the Prasiola‐clade are marked in bold. n.t., no trace of respective compound

	Prasiolin/prasiolin‐like (mg · g^−1^ DW)	d‐ribitol (mg · g^−1^ DW)	d‐sorbitol (mg · g^−1^ DW)
***Prasiococcus calcarius*** **SAG 10.95**	2.44	n.t.	75.4
***Prasiola stipitata*** **SRN 124**	12.30	15.4	31.2
***Prasiola stipitata*** **SRN 125**	21.51	11.3	27.6
***Prasiolopsis ramosa*** **SAG 26.83**	57.8	n.t.	87.1
***Trichophilus welckeri*** **SAG 84.81**	2.53	n.t.	53.8
***Rosenvingiella radicans*** **SBDN 005**	10.26	n.t.	92.0
***Rosenvingiella radicans*** **SBDN 1096A**	4.54	n.t.	101.5
***Rosenvingiella radicans*** **SRN 75**	10.51	n.t.	17.2
***Prasiola crispa*** **SAG 43.96**	7.28	n.t.	n.t.
***Prasiola calophylla***	2.67	n.t.	6.1
***Stichococcus bacillaris*** **SAG 397‐1b**	0.09	n.t.	21.0
***Pseudomarvania aerophytica*** **SAG 2148**	0.24	n.t.	44.9
***Pseudomarvania ampullaeformis*** **SAG 2047**	0.19	n.t.	21.4
***Desmococcus spinocystis*** **SAG 2067**	9.48	n.t.	31.3
***Stichococcus deasonii*** **SAG 2139**	0.55	n.t.	9.3
***Stichococcus jenerensis*** **SAG 2138**	0.08	n.t.	10.3
***Pseudochlorella signiensis*** **var.** ***communis*** **SAG 2110**	4.53	n.t.	n.t.
*Trebouxia arboricola* SAG 219‐1a	n.t.	13.8	n.t.
*Lobosphaera incisa* SAG 2007	n.t.	n.t.	n.t.
*Myrmecia bisecta* SAG 2043	n.t.	n.t.	n.t.
*Trochisciopsis tetraspora* SAG 19.95	n.t.	n.t.	n.t.
“*Stichococcus*” *mirabilis* SAG 379‐3a	n.t.	n.t.	n.t.

### MAA induction experiment

Due to their central position within the *Prasiola*‐clade (Hodač et al. 2016, Garrido‐Benavent et al. 2017), the three strains SAG 2148, SAG 2139 and SAG 379‐1d were chosen for the UV‐induction experiment. These isolates were pre‐cultivated in 100 mL Erlenmeyer flasks for 3 d under the conditions mentioned above to guarantee vital log phase cultures. Subsequently, the strains were transferred to 600 mL glass petri dishes, provided with new medium and kept at 22°C–23°C for 4 d. Additionally, two radiation conditions were applied during a 16:8 h light:dark cycle: PAR only (400–700 nm) and PAR + UVR (PAR + UV‐A + UV‐B, 295–700 nm). In both control and UV‐treatment, Lumilux Deluxe Daylight L15W/950 (OSRAM) provided 80–90 μmol photons · m^−2^ · s^−1^ PAR. UVR was emitted by Q‐Panel‐UVA 340 fluorescent lamps (Q‐Panel, Cleveland, OH, USA). While the control was covered with a 400 nm cut‐off filter foil (Folex PR; Folex, Dreieich, Germany) resulting in total UV‐A and UV‐B elimination, the UV‐treated algal cultures were exposed to 6–7 W · m^−2^ UV‐A and 0.37–0.45 W · m^−2^ UV‐B under a 295 nm cut‐off filter (Ultraphan UBT 295; Digefra, Fürstenfeldbruck, Germany). PAR was measured with a Li‐Cor LI‐190‐SB cosine corrected sensor connected to a Li‐Cor LI‐1000 data logger (Lambda Instruments, Lincoln, NE, USA). A PMA broadband radiometer (Solar Light Co., Philadelphia, PA, USA) was used to measure UVR. After the exposure period, biomass was harvested as described above. As an indicator of physiological performance, chlorophyll *a* fluorescence, that is, maximum quantum yield of photosystem II in the dark‐adapted state (F_v_/F_m_), was determined using a pulse amplitude modulated fluorometer (PAM 2500; Walz, Effeltrich, Germany) according to Graiff et al. (2015). The filters were dark incubated for 20 min at 22°C before F_v_/F_m_ was measured (*n* = 5). Finally, the filters were dried and weighed as explained above.

### MAA analysis

The dried algal samples were ground in a microdismembrator (Sartorious, Göttingen Germany) in precooled Teflon jars for 4 min at a shaking frequency of 1,800 rpm and subsequently extracted with water (100%) in an ultrasonic bath (Bandelin Sonorex 35 kHz, Berlin, Germany) for 30 min at 25°C. After centrifugation at 1,200*g* for 5 min (Heraeus Labofuge 400; Thermo Fisher, Waltham, MA, USA), the supernatant was collected and evaporated in an air stream. To guarantee exhaustive extraction, this step was repeated twice. For HPLC analysis, the combined extract was re‐dissolved in 5 mL water. MAA analysis was carried out on an Agilent 1260 HPLC system (Santa Clara, CA, USA) coupled to an amaZon iontrap mass spectrometer (Bruker, Bremen, Germany) using a Triart C18 column (150 × 3.00 mm, 3 μm) from YMC (Dinslaken, Germany). The mobile phase was comprised of 0.25% (v/v) formic acid in water (A) and 0.25% (v/v) formic acid in methanol (B). Elution was carried out in isocratic mode at 2% B for 15 min, and a gradient elution to 30% from 15 to 25 min, followed by a 10 min step of re‐equilibration. The DAD was set to 320 nm and flow rate, injection volume and column thermostat were adjusted to 0.3 mL · min^−1^, 5 μL and 30°C, respectively. Sample quantification was carried out using HPLC‐UV, but for most samples LC‐MS experiments were additionally performed. MS‐spectra were recorded in positive ESI mode, with a drying gas temperature of 200°C, the nebulizer gas (nitrogen) set to 23 psi, and a nebulizer flow (nitrogen) of 8 L · min^−1^.

For the quantitative determination of MAAs, a calibration curve for prasiolin was established (regression equation: *Y* = 5.085 *x* − 2.3887; determination coefficient = 0.9999; linear range = 1.15 to 147.6 μg · mL^−1^). The second MAA (“prasiolin‐like”) was quantified accordingly.

### Polyol analysis

At least 10 mg DW of both cultured and field sampled algae were used. The material was placed in screw‐capped centrifugation tubes filled with 1 mL 70% ethanol (HPLC‐grade, v/v) and kept in a water bath at 70°C for 4 h. For higher extraction success, the tubes were vortexed occasionally. After centrifugation at 13,000*g* for 5 min, 800 μL of the supernatant were transferred to a new vial and evaporated to dryness under vacuum (Savant SpeedVac SVC 100H; Thermo Fisher Scientific). The pellets were re‐dissolved in 800 μL ddH_2_O (HPLC‐grade) and centrifuged at 13,000*g* for 5 min. The supernatant was transferred to a HPLC‐vial. For analysis, an Agilent 1260 Infinity Series HPLC system (Agilent) equipped with a vacuum degasser, a quaternary pump, a refractive index detector and a Fast Carbohydrate Analysis Column (Bio Rad, Hercules, CA, USA) was used. Samples were eluted in ddH_2_O (HPLC‐grade) at a flow rate of 1 mL · min^−1^ at 70°C according to Karsten et al. (1991).

### Phylogenetic analysis

All phylogenetic analyses are based on 18S rRNA gene sequences originating from GenBank, where all sequences of SAG strains are publicly available. For the new isolates of *Prasiola stipitata*,* P. calophylla* and *Rosenvingiella radicans*, the sequence of the most similar respective relative was chosen (for full list of organisms and accession numbers see Table S1 ). To obtain a tree that coincides with latest Trebouxiophyceae‐phylogenies, additional sequences were chosen from Hallmann et al. (2016). Multiple alignments were conducted with the Muscle algorithm implemented in MEGA version 7 (Kumar et al. 2016). Based on the lowest AIC (Akaike 1981) calculated with jModelTest implemented in MEGA version 7 (Kumar et al. 2016), the best evolutionary model for the data set was chosen. The phylogenetic tree was constructed with the program MrBayes 3.2.2 (Ronquist et al. 2012), using the GTR+Γ+I model with 5,000,000 generations. Two out of four runs of Markov chain Monte Carlo were made simultaneously, with trees taken every 500 generations. Split frequencies between runs at the end of calculations were below 0.01. The trees selected before the likelihood rate reached saturation were subsequently discarded. Finally, branches were collapsed for clarity if the taxa were needed to obtain a stable tree but were not further investigated.

### Statistical analysis

Homoscedastic, independent *t*‐tests were conducted with R version 3.4.0 (R‐Development‐Core‐Team 2017). Based on the biological background and the hypotheses, a two‐tailed *t*‐test was performed for the comparison of the maximum quantum yields, whereas a one‐tailed *t*‐test was used for the comparison of the prasiolin contents.

## Results

### Phylogenetic analysis

**Figure 1:**
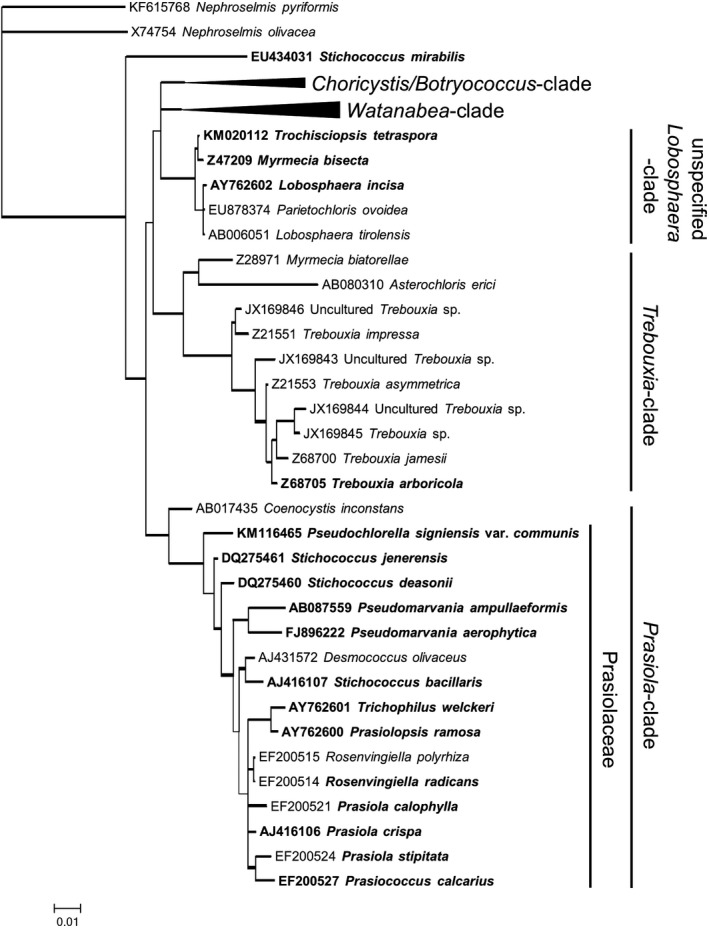
Maximum likelihood (ML) phylogeny based on 18S rRNA gene sequences of investigated Trebouxiophyceae as well as strains taken from Hallmann et al. (2016). Thick branches indicate an ML bootstrap support ≥0.9. Bold names indicate investigation in this study. The scale bar corresponds to 0.01 substitutions per site.

To visually support the interpretation of the results, a phylogenetic tree including the 22 investigated Trebouxiophyceae was derived from 18S rRNA gene sequences. The sequences of the *Choricystis*/*Botryococcus*‐ and *Watanabea*‐clade were needed to obtain a stable tree. However, no member of these taxa was investigated in this study, which is why the branches were collapsed for more clarity (Fig. 1). The tree mainly coincides with the phylogeny presented in Hallmann et al. (2016) and Garrido‐Benavent et al. (2017). Moreover, “*Stichococcus*” *mirabilis* SAG 379‐3a is located outside the *Prasiola*‐clade, as already shown in Mikhailyuk et al. (2008).

### Polyol and MAA analysis

**Figure 2:**
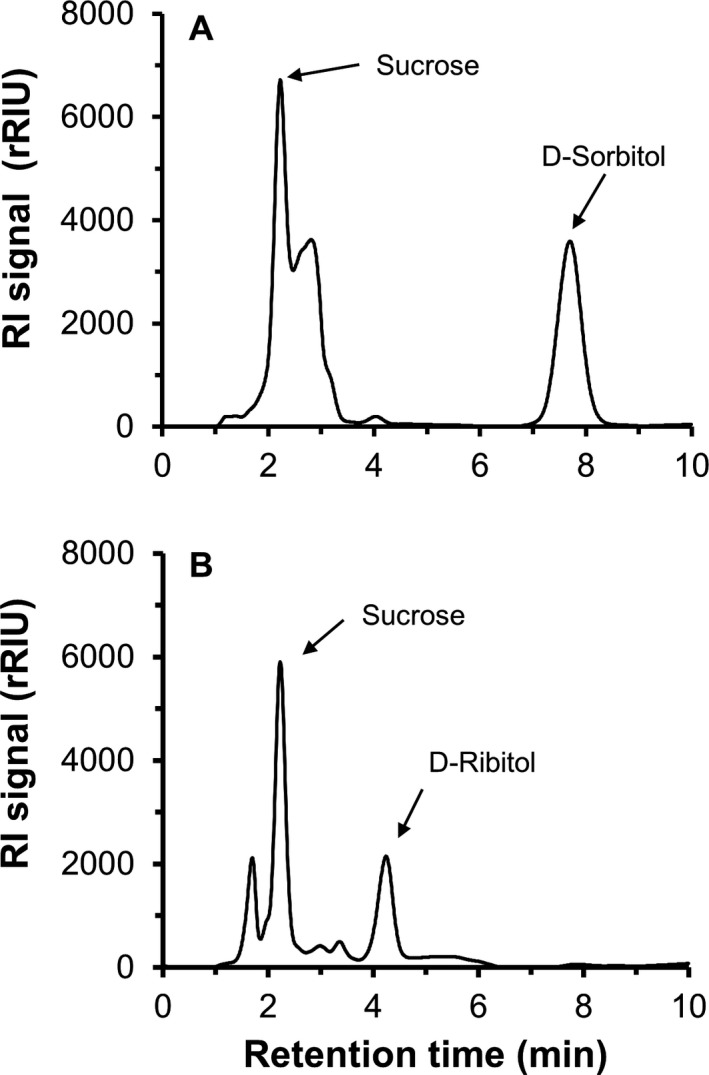
HPLC chromatograms of ethanolic extracts of (A) *Rosenvingiella radicans* and (B) *Trebouxia arboricola*. While the *R. radicans* extract shows a high d‐sorbitol peak at 6.7 min, a d‐ribitol peak at 4.2 min was detected in *T. arboricola*. Abbreviations: RI signal, Refractive Index signal; rRIU, relative Refractive Index Units.

The polyols d‐sorbitol and d‐ribitol were present in 16 of the 22 studied aeroterrestrial Trebouxiophyceae (Table 1). An exemplary chromatogram for both compounds is shown in Figure 2. Except for *Prasiola crispa* and *Pseudochlorella signiensis* var. *communis*, d‐sorbitol was found in all tested members of the *Prasiola*‐clade (Hallmann et al. 2016): *Prasiococcus calcarius*,* P. stipitata*,* P. calophylla*,* Rosenvingiella radicans*,* Prasiolopsis ramosa*,* Trichophilus welckeri*,* Pseudomarvania aerophytica*,* Pseudomarvania ampullaeformis*,* Stichococcus bacillaris*,* Stichococcus deasonii*,* Stichococcus jenerensis* and *Desmococcus spinocystis*. Concentrations ranged from ~9 mg · g^−1^ DW in *S. deasonii* to over 100 mg · g^−1^ DW in *R. radicans* (SBDN 1096A). Field material of *R. radicans* (SRN75) contained less d‐sorbitol than cultivated material (SBDN 1096A, SBDN 005) by ~6‐fold. Around 30 mg · g^−1^ DW of d‐ribitol were detected in both field samples of *P. stipitata* (*Prasiola*‐clade) as an additional polyol to d‐sorbitol (Table 1). In the phylogenetically distinct *Trebouxia arboricola*, d‐ribitol was the only present polyol at a concentration of ~14 mg · g^−1^ DW. Neither d‐sorbitol nor d‐ribitol was detected in *P. crispa*,* P. signiensis* var. *communis*,* Lobosphaera incisa*,* Myrmecia bisecta*,* Trochisciopsis tetraspora* and “*S*.”* mirabilis* (Table 1). The HPLC chromatogram of *P. crispa* did not show any polyol peak, indicating that the used material was in a degenerated physiological state and hence no polyols could be extracted.

**Figure 3:**
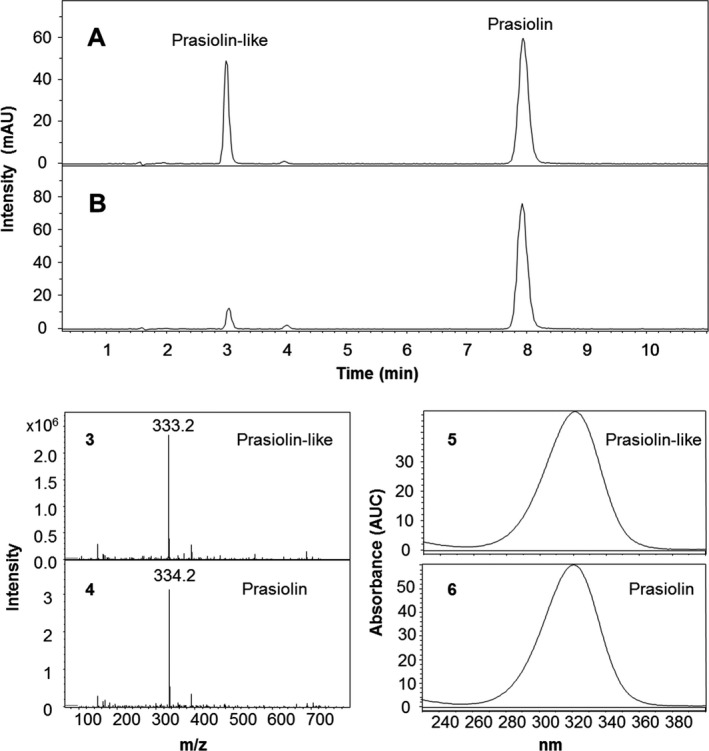
Analytical results for *Prasiola calophylla*. (A) LC‐MS chromatogram of a freshly prepared aqueous extract and (B) the same extract analyzed after 24 h. The segments below show the MS‐ (3) and UV spectra (5) of the Prasiolin‐like constituent, (4) and (6) the corresponding data for Prasiolin. MS spectra were recorded in positive ESI mode.

After preliminary experiments concerning the optimum extraction protocol, all strains were additionally analyzed for the presence of the MAA prasiolin. These experiments were carried out using *Prasiola calophylla*, the strain from which prasiolin was originally isolated. They showed that after grinding the cells in a dismembrator, a threefold extraction for 30 min each using water as solvent is exhaustive. To account for the instability of prasiolin, the temperature in the sonicator was kept at 25°C by constantly adding ice, and evaporation of the solvent was carried out under cold airstream instead of using a rotary evaporator. An interesting observation was made when using pure methanol for extraction, because instead of prasiolin (Mr = 333) another MAA with an identical UV‐spectrum but a molecular mass of only 332 was found. In aqueous extracts (100% water and 25% methanol), both peaks appear, “prasiolin‐like” at 3.1 min and prasiolin at 8.0 min (Fig. 3). Both compounds must be of highly similar structure, because they also convert into each other; after 24 h the peak area of “prasiolin‐like” declines and that of prasiolin increases to the same extent. After 48 h none of the two MAAs is detectable anymore. It can be hypothesized that “prasiolin‐like” may contain a glutamine residue instead of glutamic acid at the nitrogen in position 3, because this would explain the mass difference of 1 Da. The isolation of “prasiolin‐like,” even though it is challenging due to instability reasons, is currently in progress. For this study, both MAAs were quantified together as sum using the calibration data of prasiolin (Fig. 3), and their presence/absence is shown in Table 1. Most interesting is the observation that prasiolin could only been detected in *Prasiola calophylla* and both *P. stipitata* samples, all of which were collected in the field. In all other algal strains tested, which originated from a culture collection, “prasiolin‐like” was the only MAA present (Table 1).

Prasiolin and/or “prasiolin‐like” were found in all tested members of the *Prasiola*‐clade, with concentrations ranging from as low as 10 μg · g^−1^ DW (*S. bacillaris*) to ~58 mg · g^−1^ DW (*P. ramosa* SAG 26.83). Outside the *Prasiola*‐clade, no MAAs were detected (Table 1).

### MAA induction experiment

**Figure 4:**
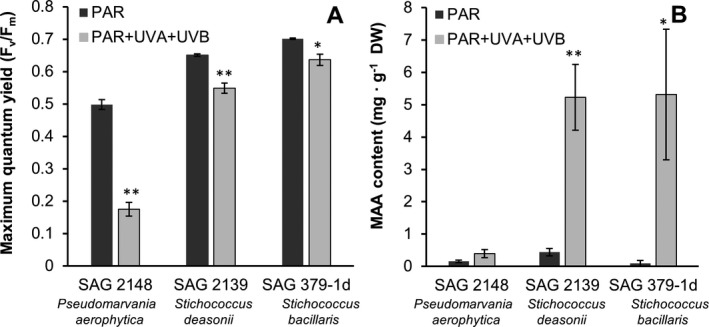
Results of the MAA induction experiment under UV radiation conditions for three SAG strains from the *Prasiola*‐clade. (A) Maximum quantum yield of PSII and (B) prasiolin/“prasiolin‐like” content in mg · g^−1^ DW after four d of PAR and UVR exposure, respectively (*n* = 3). Error bars indicate standard errors. *P < 0.05, **P < 0.01.

To evaluate whether the UV‐absorbing MAAs are inducible and accumulate under controlled UVR, UV exposure experiments were conducted with *Prasiola aerophytica*,* Stichococcus deasonii* and *S. bacillaris* (Fig. 4). Prior to MAA extraction, the maximum quantum yield of photosystem II in the dark‐adapted state (F_v_/F_m_) was determined (Fig. 4A). In *Prasiola aerophytica* an F_v_/F_m_ value of 0.50 was measured under control conditions, which decreased to 0.18 after UV treatment. In *S. deasonii* an F_v_/F_m_ value of 0.65 was detected in the control, which slightly dropped to 0.55 under UV exposure. In the *S. bacillaris* control, the maximum quantum yield was 0.70, and UV exposure led to a minor reduction to 0.64 (Fig. 4A). Compared to the respective control, both *P. aerophytica* and *S. deasonii* showed a significantly lower F_v_/F_m_ in the UV treatment (*P* < 0.01). Maximum quantum yield in *S. bacillaris* was also significantly reduced (*t*‐test, *t*_4_ = 6.213, *P* < 0.05) under UVR compared to PAR (Fig. 4A).

MAA inducibility and accumulation due to UV exposure was observed in all three strains (Fig. 4B). In both *Stichococcus deasonii* and *S. bacillaris* the amount of prasiolin like compounds significantly increased from less than 0.5 mg · g^−1^ DW in the control to ~5 mg · g^−1^ DW in the UV treatment (*t*‐test, *t*_4_ = −4.689, *P* < 0.01 [*S. deasonii*] and *t*_4_ = −2.583, *P* < 0.05 [*S. bacillaris*]). Although *Prasiola aerophytica* doubled its MAA content from less than 0.16 mg · g^−1^ DW to ~0.3 mg · g^−1^ DW, this increase was not significant (*t*‐test, *t*_4_ = −1.836, *P* > 0.05; Fig. 4B).

## Discussion

The morphological similarity and high phenotypic plasticity of green microalgae (Rindi and Guiry 2002, Rindi 2007, Darienko et al. 2015, 2016) makes their taxonomy a challenging task (John and Maggs 1997, Rindi 2007). Moreover, in the modern molecular age of science, morphology gets much less attention compared to former times. Instead, many authors follow polyphasic approaches in which morphological data are combined with those derived from ecophysiology and cell biology as well as various molecular markers (e.g., Darienko et al. 2010). In addition, the discovery of suitable chemotaxonomic markers might be very useful in green microalgal taxonomy, but so far only few studies have been published on this topic (e.g., Gustavs et al. 2011, and references therein). Chemotaxonomic characters such as polyol and MAA patterns are particularly helpful when sequence information is not available or questionable. Additionally, both polyol and MAA analyses are quite easy and quick to undertake in the lab and provide a chemical fingerprint as part of a polyphasic approach.

For this study, 22 aeroterrestrial Trebouxiophyceae were chemically screened for the presence of the polyols d‐sorbitol and d‐ribitol as well as MAAs, with a focus on relatives of *Prasiola calophylla*. Additionally, a phylogenetic tree based on 18S rRNA gene sequences was calculated (Fig. 1). It strongly resembles the phylogeny presented by Hallmann et al. (2016) and Garrido‐Benavent et al. (2017). Moreover, the *Prasiola*‐clade matches the distribution of d‐sorbitol and prasiolin/“prasiolin‐like” within the Trebouxiophyceae: The polyol d‐sorbitol was exclusively found in members of the *Prasiola*‐clade (Table 1; Roser et al. 1992, Gustavs et al. 2011). The compound is absent only in one of its tested members: *Prasiola signiensis* var. *communis*. According to Garrido‐Benavent et al. (2017), the 16 positively tested strains belong to the family Prasiolaceae, whereas *P. signiensis* var. *communis* does not (Darienko et al. 2016). This circumstance indicates that the distribution of d‐sorbitol within the *Prasiola*‐clade is restricted to the family Prasiolaceae. Gustavs et al. (2011) chemically investigated the presence of various polyols in 34 mainly aeroterrestrial Trebouxiophyceae belonging to 5 different clades. The results of these authors are in accordance with this study. Considering the obvious phylogenetic distribution of d‐sorbitol within aeroterrestrial Trebouxiophyceae (Table 1), these findings provide strong evidence for d‐sorbitol as a suitable chemotaxonomic marker for the family Prasiolaceae.

Based on Karsten et al. (2005) and Hartmann et al. (2016), the 324 nm MAA prasiolin was expected in all relatives of *Prasiola calophylla*. Indeed, in all members of the *Prasiola*‐clade either prasiolin and/or a “prasiolin‐like” MAA was detected (Table 1). As mentioned before, prasiolin was only found in field samples of *P. calophylla* and *P. crispa*, while the “prasiolin‐like” compound dominated all other algal strains, which were provided by the SAG culture collection. From these data, it might be possible to assume that natural environmental conditions with usually high insolation stimulate the prasiolin formation and accumulation, while long‐term cultivation under artificial light rather leads to the production of the “prasiolin‐like” compound.

Furthermore, this study coincides with the findings of Bandaranayake (1998) and Karsten et al. (2005), although the putative 324 nm MAA mentioned in these earlier publications was structurally confirmed as prasiolin just recently (Hartmann et al. 2016). The 324 nm MAA was also found in two members of the *Watanabea*‐clade (Karsten et al. 2005), and most probably represents prasiolin or “prasiolin‐like,” too, but chemical verification using MS and NMR techniques (Hartmann et al. 2016) is still missing. The presence of this 324 nm MAA in unknown green algal specimens can be used chemotaxonomically, as they can either be assigned to the *Watanabea*‐ or to the *Prasiola*‐clade (Karsten et al. 2005). However, the occurrence of prasiolin/“prasiolin‐like” only allows for the exclusion of taxa that lack these MAAs and thereby to confine the remaining relationship‐possibilities, rather than the assignment to one specific clade based on the sole presence of these UV‐sunscreens.

Altogether, the phylogenetic tree (Fig. 1) emphasizes the exclusive occurrence of d‐sorbitol and prasiolin‐like compounds in the Prasiolaceae and *Prasiola*‐*clade*, respectively. “*S*.”* mirabilis* contains neither d‐sorbitol, nor prasiolin/“prasiolin‐like” (Table 1), which supports the position of “*S*.”* mirabilis* outside the *Prasiola*‐clade as already shown in Mikhailyuk et al. (2008). A re‐evaluation of this species should be considered, as both the polyol and MAA content as well as the phylogenetic position derived from 18S rRNA gene sequence show that “*S*.”* mirabilis* does not belong to the genus *Stichococcus*.

The polyol d‐ribitol was unexpectedly detected in two independent *Prasiola stipitata* strains. Even though this polyol is widely distributed, for instance in members of the *Watanabea*‐, *Elliptochloris*‐ and *Trebouxia*‐clade (Maruo et al. 1965, Richardson and Smith 1968, Gustavs et al. 2010, 2011, Sadowsky et al. 2016), it has not yet been reported to be present in any member of the *Prasiola*‐clade. Moreover, Gustavs et al. (2011) examined *P. stipitata* field material from Germany, but only found d‐sorbitol. Field material is known to be prone to contamination, such as epiphytic algae. Since the two *P. stipitata* field samples for this study were microscopically examined to exclude contamination prior to HPLC analysis, and because the detected d‐ribitol concentrations were of the same order of magnitude as the second polyol, d‐sorbitol, it is highly reasonable to assume that d‐ribitol is indeed synthesized by these *P. stipitata* strains. The presence of a set of polyols has been interpreted as a biochemical trait to better cope with fluctuating environmental stress factors that come along with a terrestrial lifestyle (Gustavs et al. 2011). However, under osmotic and matric stress, *P. crispa* ssp. *antarctica* and a phylogenetically related *Stichococcus* species (Prasiolaceae) only accumulated d‐sorbitol (Jacob et al. 1991, Gustavs et al. 2010). The occurrence of d‐ribitol as an additional polyol in both *P. stipitata* strains suggests a unique biochemical capability of this species within the genus *Prasiola*. To confirm this hypothesis, however, further ecophysiological studies on *P. stipitata* are required. Nevertheless, these findings are a first hint that this particular *Prasiola*‐species has additional biochemical traits that are missing in close relatives.

One hypothesis of this study was that MAAs like prasiolin are UV‐inducible. This seems to be the case only for *Stichococcus bacillaris* and *S. deasonii* (Fig. 4B). In contrast to both *Stichococcus*‐species, the average maximum quantum yield (Fig. 4A) in the *Prasiola aerophytica* control was at least 30% lower than previously reported literature values (Juneau and Harrison 2005, Gray et al. 2007, Kang et al. 2013, Guéra et al. 2016, Zhang et al. 2017), indicating that the applied cultivation methods were not suitable for this species. Hence, the physiological performance in *P. aerophytica* was already negatively affected under control conditions. UV exposure led to a slight decrease of the F_v_/F_m_ in both *Stichococcus* strains (Fig. 4A). A similar response was observed in an unspecified *Stichococcus*‐species isolated from a building façade (Karsten et al. 2007b). The maximum quantum yield in *P. aerophytica*, however, severely decreased after the UV treatment (Fig 4A). The MAA content in both control and UVR exposed algae mirrored these findings, as only a minor accumulation was observed (Fig. 4B): As MAAs are UV protectants (Bandaranayake 1998), the strong increase in MAAs in both *Stichococcus* strains explains the relatively low impact of UVR on their maximum quantum yield. Conversely, the low MAA content in *P. aerophytica* might be a reason for the low maximum quantum yield after UV exposure. Nevertheless, considering the general presence of prasiolin and/or “prasiolin‐like” in *P. aerophytica* (Table 1; Fig. 4B) and its phylogenetic position within the *Prasiola*‐clade, physiologically unaffected *P. aerophytica* is most likely capable of MAA accumulation, too. Nevertheless, the prasiolin/“prasiolin‐like” MAAs were shown to be inducible under UVR. Thereby, the results of this part of the presented study are in accordance with Karsten et al. (2007b) and additionally prove that prasiolin/“prasiolin‐like” are UV‐inducible, and most probably UV‐protective substances. Moreover, these findings provide new evidence that the distribution of these MAAs is not only attributable to phylogenetic relations, but also to ecophysiological acclimation.

The main goal of this study was to emphasize the value of easily detectable chemical traits in green microalgal taxonomy. Furthermore, we aimed to establish the polyol d‐sorbitol and prasiolin/“prasiolin‐like” as suitable chemotaxonomic markers for the Prasiolaceae and *Prasiola*‐clade, respectively. Both are highly abundant taxa of aeroterrestrial green algae with a worldwide distribution, but also a challenging taxonomy. Especially d‐sorbitol was proven to be of high chemotaxonomic value, as it is exclusively found in the Prasiolaceae‐family. Prasiolin/“prasiolin‐like” compounds are not only present in the *Prasiola*‐clade, but also in the *Watanabea*‐clade (Karsten et al. 2005), and thus have a wider distribution amongst the Trebouxiophyceae than d‐sorbitol. Nevertheless, they still are a suitable chemotaxonomic marker, because their presence reduces the remaining possible affiliations down to either the *Prasiola*‐, or the *Watanabea*‐clade. Since aeroterrestrial green algae are taxonomically highly diverse, with many taxa belonging to the Chlorophyta and Streptophyta, this study is the first to simultaneously establish two groups of chemically independent chemotaxonomic markers, a polyol and MAAs, for the *Prasiola*‐clade. In addition, further investigations on this highly useful and promising topic are required to better understand the protective functions of both metabolites in these terrestrial green algae.

The authors deeply thank Dr. Svenja Heesch and Prof. Andreas Holzinger for providing algal samples. This study was financially supported by the Deutsche Forschungsgemeinschaft (DFG; KA899/16) to U.K. and the Austrian Science Fund (FWF; ZFP296710) to M.G.

## Supporting information


**Table S1.** List of algae used for phylogenetic analysis.
