# Supplementary material for: Polyols and UV‐sunscreens in the Prasiola‐clade (Trebouxiophyceae, Chlorophyta) as metabolites for stress response and chemotaxonomy
Source: J Phycol. 2018 Feb 21;54(2):264–74. doi: 10.1111/jpy.12619 (PMC5947255; doi:10.1111/jpy.12619)
Supplement: Supplementary file 1 — Table S1. List of algae used for phylogenetic analysis. [file JPY-54-264-s001.docx]

**Table S1** List of algae used for phylogenetic analysis.

| **Strain/isolate** | **Name** | **Accession number** |
| --- | --- | --- |
| CCMP717 | *Nephroselmis pyriformis* | KF615768 |
|  | *Nephroselmis olivacea* | X74754 |
| Prca1 | *Prasiococcus calcarius* | EF200527 |
| SAG 43.96 | *Prasiola crispa* | AJ416106 |
| Psti1 | *Prasiola stipitata* | EF200524 |
| Pcal1 | *Prasiola calophylla* | EF200521 |
| Rrad1 | *Rosenvingiella radicans* | EF200514 |
| Rpol1 | *Rosenvingiella polyrhiza* | EF200515 |
| SAG 26.83 | *Prasiolopsis ramosa* | AY762600 |
| SAG 84.81 | *Trichophilus welckeri* | AY762601 |
| CAUP H 7301 | *Pseudomarvania aerophytica* | FJ896222 |
| SAG 2047 | *Pseudomarvania* *ampullaeformis* | AB087559 |
| SAG 397-1b | *Stichococcus bacillaris* | AJ416107 |
| SAG 2139 | *Stichococcus* *deasonii* | DQ275460 |
| SAG 2138 | *Stichococcus jenerensis* | DQ275461 |
| SAG 35.83 | *Desmococcus* *olivaceus* | AJ431572 |
| SAG 2110 | *Pseudochlorella* *signiensis* var. *communis* | KM116465 |
| SAG 219-1a | *Trebouxia* *arboricola* | Z68705 |
|  | *Lobosphaera* *tirolensis* | AB006051 |
| ACKU 177-03 | *Parietochloris* *ovoidea* | EU878374 |
| SAG 2007 | *Lobosphaera* *incisa* | AY762602 |
| SAG 2043 | *Myrmecia* *bisecta* | Z47209 |
| SAG 19.95 | *Trochisciopsis* *tetraspora* | KM020112 |
| SAG 379-3a | *Stichococcus mirabilis* | EU434031 |
| UBT-86.132E2 | *Trebouxia jamesii* | Z68700 |
|  | *Trebouxia* *impressa* | Z21551 |
|  | *Trebouxia* *asymmetrica* | Z21553 |
| GOGs_18S_K02 | Uncultured *Trebouxia* sp. | JX169843 |
| GOGre_K46 | *Trebouxia* sp. | JX169845 |
| GOGsk_K6 | Uncultured *Trebouxia* sp. | JX169844 |
| GOGsM_K51 | Uncultured *Trebouxia* sp. | JX169846 |
|  | *Asterochloris* *erici* | AB080310 |
| UTEX 907 | *Myrmecia* *biatorellae* | Z28971 |
|  | *Coenocystis* *inconstans* | AB017435 |
| SAG 211-9b | *Watanabea* *reniformis* | X73991 |
| SAG 3.95 | *Chloroidium* *ellipsoideum* | FM946012 |
| SAG 211-9a | *Chlorella* *saccharophila* | FM946000 |
| SAG 2213 | *Heterochlorella luteoviridis* | KM116462 |
| SAG 2037 | *Apatococcus* *lobatus* | JX169825 |
| SAG 2359 | *Apatococcus* *lobatus* | JX169826 |
| SAG 2036 | *Symbiochloris* *irregularis* | GU017659 |
| SAG 216-13 | *Coccomyxa* *subellipsoidea* | HG972978 |
| SAG 10.92 | *Viridiella fridericiana* | FM958481 |
| ‘Titicaca’ | *Botryococcus* *braunii* | AJ581912 |
| UTEX 274 | *Pseudococcomyxa* *simplex* | FJ648514 |
| SAG 2117 | *Elliptochloris* sp. | FJ648515 |
| SAG 245.80 | *Elliptochloris* *bilobata* | AM422984 |
